# Phytochemical analysis and antioxidant, antimicrobial, and cytotoxic activities of *Xanthium strumarium* L. (Asteraceae)

**DOI:** 10.55730/1300-0152.2730

**Published:** 2024-12-16

**Authors:** Ahmet BEYATLI

**Affiliations:** 1 Medicinal and Aromatic Plants Program, Hamidiye Vocational School of Health Services, University of Health Sciences, İstanbul, Turkiye; 2 School of Agriculture, Food and Ecosystem Sciences, Faculty of Science, Food Science and Nutrition, University of Melbourne, Melbourne, Australia

**Keywords:** *Xanthium strumarium*, antioxidant, antimicrobial, cytotoxicity, HPLC-DAD

## Abstract

**Background/aim:**

Medicinal plants are considered an important source of novel antioxidant, antimicrobial, and anticancer agents. The main goal of this study was to define the beneficial properties of various extracts obtained from *Xanthium strumarium*.

**Materials and methods:**

Evaluations were conducted on the total phenolic and flavonoid contents of the extracts. High-performance liquid chromatography and diode-array detection (HPLC-DAD) was used to determine the phenolic profiles of the extracts. DPPH, ABTS, and FRAP assays were used to evaluate the free radical scavenging properties of the *X. strumarium* extracts. The broth dilution method was used for antimicrobial activity assessments, the MTT assay was used for the evaluation of cytotoxicity in K562 and P3HR1 cells treated with the extracts, and western blotting was used for the determination of molecular pathways. DNA fragmentation was also conducted utilizing the diphenylamine assay.

**Results:**

The total phenolic and flavonoid contents of the acetone extract were significantly higher than those of the methanol and ethanol extracts at 454.54 mg GAE/g and 78.94 mg catechin/g, respectively. The acetone extract had the highest amounts of flavonoids. All extracts exhibited noTable antioxidant activity. The acetone extract had lower minimum inhibitory concentrations than the other extracts against the studied bacterial and fungal strains. The extracts showed varying degrees of cytotoxicity in the studied cell lines and all such effects were dose-dependent and solvent-specific. Half-maximal inhibitory concentration (IC_50_) values ranged between 180.12 and 410.23 μg/mL, with the lowest IC_50_ value being obtained for the acetone extract. Treatments led to cytochrome c release and high expression of caspase-3 and caspase-8, which can be attributed to the involvement of mitochondria in the process of apoptosis. The DNA fragmentation percentage increased in both cell lines with all extracts.

**Conclusion:**

Based on these findings, *X. strumarium* demonstrates significant antioxidant, antimicrobial, and anticancer properties. Notably, the acetone extract exhibited the strongest activity across the tested parameters, highlighting its potential for further pharmaceutical and therapeutic applications.

## Graphical Abstract

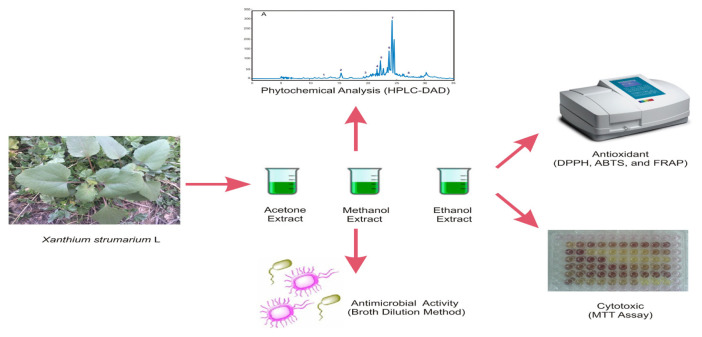

## 1. Introduction

Medicinal plants are a reliable source of compounds with medicinal properties. In addition to being widely studied, many plants are of considerable economic interest because they are widely used in agriculture, pharmaceuticals, and cosmetics due to the wide variety of antioxidant, antimicrobial, and anticancer compounds that they contain (Cragg and Newman, 2005; Akhtar et al., 2018).

Various pathogenic microorganisms are associated with a wide range of diseases. Skin infections, abscesses, pneumonia, and potentially fatal illnesses like sepsis are all frequently brought on by *Staphylococcus aureus*. Endocarditis, intraabdominal infections, and urinary tract infections (UTIs) can all be caused by *Enterococcus faecalis*. While some strains of *Escherichia coli* cause hemolytic-uremic syndrome and gastroenteritis, uropathogenic strains in particular are a major cause of UTIs. Particularly in immunocompromised individuals, *Pseudomonas aeruginosa* is well known for causing hospital-acquired infections such as bacteremia, wound infections, and pneumonia. Finally, the fungal pathogen *Candida albicans* is the cause of candidiasis, with manifestations ranging from systemic infections in patients with impaired immune systems to superficial infections like oral thrush (Murray et al., 2020). Infectious diseases and microbial pathogenicity were treated with natural products for thousands of years worldwide, prior to the introduction of antibiotics and other modern medications (Mohr, 2016). Bacterial infection is considered one of the significant contributors to human illness in both developed and developing countries. These pathogens evolve with time and gain resistance to formerly discovered antibiotics (Chokshi et al., 2019).

Cancer is a disease in which cells grow, differentiate, and die at excessive and uncontrolled rates. There are more than 100 different clinical pathologies associated with cancer, rendering it one of the top causes of mortality in many nations (Kufe et al., 2003). Leukemia is a malignancy that is defined as a set of diverse neoplastic disorders of white blood cells characterized by excessive growth and a halt in hematopoietic cell differentiation (Lee et al., 2007). Leukemia treatment requires an interdisciplinary approach. Chemotherapy, immunological therapy, hormone therapy, radiotherapy, and symptomatic and supportive therapy can be listed as the main therapeutic options. The main difficulties in the treatment of cancer are drug resistance, toxicity, and low specificity. Drug resistance particularly necessitates the quest for novel medications, and medicinal plants are one of the most promising possible sources. In addition, a huge portion of the global population lacks access to traditional pharmaceutical treatment. Natural materials might thus be considered one of the best accessible alternatives to modern medications in cancer therapy because of their low cost and toxicity (Rates, 2001). Many plant-derived bioactive compounds have been found and commercialized as cancer therapies, including camptothecin, paclitaxel, and vinblastine, which are derived from *Camptotheca acuminata*, *Taxus brevifolia*, and *Catharanthus roseus*, respectively (Vickers, 2002). Antileukemic medicines have been discovered from medicinal plants, which have proven to be effective natural sources. The cytotoxicity of these plants, the mechanisms underlying their toxicity for leukemic cells, and their isolated compounds have been intensively studied in recent years. Significant advancements have been made and significant milestones have been reached in leukemia therapeutics based on plant-derived chemicals and crude extracts from various medicinal plants. Furthermore, understanding the mechanisms of action of these plants is a critical component of the whole picture (Maher et al., 2021).

*X. strumarium* L., popularly called “rough cocklebur,” “common cocklebur,” or “clotbur,” can be found all over the world but is most prevalent in the temperate zone (Löve and Dansereau, 1959). Traditional medicine particularly uses the leaves, roots, and fruits of *X. strumarium*. Constituents such as sesquiterpene lactones (xanthatin, xanthinosin, xanthanol, and isoxanthanol), glycosides, phenols, polysterols, fatty acids, carboxyatractyloside, caffeoylquinic acids, hydroquinone, and thiazinedione found in various parts of the plant explain the majority of its pharmacological effects (Han et al., 2007; Ramírez-Erosa et al., 2007; Kamboj and Saluja, 2010; Wahab et al., 2012). Smallpox, cancer, leukoderma, epilepsy, excessive salivation, malaria, rheumatism, tuberculosis, and bacterial and fungal infections have all been traditionally treated with various parts of this plant (Chakravarty, 1976). Scientific studies conducted on different crude extracts or isolated compounds from different parts of *X. strumarium* showed anticancer (Roussakis et al., 1994; Kim et al., 2003; Ramírez-Erosa et al., 2007; Sridharamurthy et al., 2011; Takeda et al., 2011; Chandel and Bagai, 2012), antiinflammatory (Yadava and Jharbade, 2007; Nibret et al., 2011), antimicrobial (Jawad et al., 1988; Srinivas et al., 2011), antioxidant (Kang et al., 2003), antiplasmodial (Le Tran et al., 2003; Chandel and Bagai, 2012; Chandel et al., 2012), antitrypanosomal (Talakal et al., 1995), and antiulcerogenic (Favier et al., 2005) activities. The major goal of the present study was to evaluate the antioxidant, antimicrobial, and cytotoxic effects of *X. strumarium* by using different solvents to prepare extracts from the plant’s roots.

## 2. Materials and methods

### 2.1. Plant materials and extraction

Common roots of *X. strumarium* were collected during June and July 2023 from wild plants growing around the town of Amerli in Salah Al-Din Governorate, Iraq (34°39′52.1″N, 44°29′05.7″E). The plant was identified and authenticated by Selçuk Tuğrul Körüklü (Department of Biology, Ankara University, Ankara, Türkiye) and a voucher specimen (ANK-60506) was deposited in the Ankara University Herbarium. A previously described procedure was used to prepare *X. strumarium* extracts (Abdel Barry, 2000). Shade-dried plant materials were ground utilizing an electric grinder to make a fine powder. In 500 mL of acetone, methanol, and ethanol, 50 g of powder was suspended and the mixture was stirred overnight at 40 °C. The suspensions were then filtered and concentrated by rotary evaporator at 40 °C.

### 2.2. Preliminary phytochemical screening

Standard techniques were used to qualitatively test the *X. strumarium* extracts for the presence of different phytochemicals (Tyler, 1993; Harborne, 1998). The results were categorized as either positive (+) or negative (−).

### 2.3. Determination of total phenolic content (TPC)

The Folin–Ciocalteu method was used to evaluate TPC in accordance with a previously described procedure (Zhou et al., 2004). The absorbance of the mixture was measured at 765 nm for both standards and samples after the mixture was allowed to sit in the dark for 30 min. Plotting the absorbance values for various concentrations of gallic acid (GAE) helped in the creation of the calibration curve. Values of TPC were expressed as mg GAE/g for each sample using the sample’s absorbance in the calibration equation.

### 2.4. Determination of total flavonoid content (TFC)

For determination of TFC in extracts or catechin, samples of 0.25 mL were mixed with 75 mL of 5% NaNO_2_ solution and stored for 5 min. Subsequently, 10% AlCl_3_·6H_2_O was added at 150 μg/L. After 5 min, 0.5 mL of 1 M NaOH was added to the mixture with absorbance at 510 nm. TFC was given as mg catechin/g sample (Dewanto et al., 2002).

### 2.5. HPLC-DAD analysis of phenolic compounds

Analysis of the phenolic compounds was conducted by high-performance liquid chromatography and diode-array detection (HPLC-DAD) using the Agilent 1200 system (Agilent Technologies, Santa Clara, CA, USA). The separation process was conducted using a Waters XBridge C18 column (3.5 μm, 4.6 × 250 mm; Waters Corp., Milford, MA, USA) maintained at 25 °C with flow rate of 0.5 mL/min and injection volume of 10 μL. The mobile phase gradient included 0.1% formic acid in water and acetonitrile as solvents A and B, respectively. The gradient elution process was as follows: 10%–13% B for 0–10 min, increasing to 41.5% B for 10–20 min and then 70% B for 20–25 min, and reverting to 10% B for 25–35 min. Agilent HPLC Chem-Station software (Agilent Technologies) was used for data processing. Wavelength measurements were performed for protocatechuic acid, epigallocatechin gallate, epicatechin gallate, vanillic acid, and naringenin at 280 nm; for resveratrol at 306 nm; for luteolin at 340 nm; and for rosmarinic acid, rutin, chlorogenic acid, caffeic acid, p-coumaric acid, kaempferol, and ferulic acid at 360 nm. The retention times and UV spectra were compared to reference standards in order to characterize the peaks.

### 2.6. In vitro antioxidant activity

The antioxidant activities of the extracts were assessed by DPPH (2,2-diphenyl-1-picrylhydrazyl) and ABTS (2,2-azino-bis-3-ethylbenzothiazoline-6-sulfonic acid) assays, which reflect free radical scavenging mechanisms (Şahin et al., 2012), and by FRAP (ferric reducing/antioxidant power) assay for transition metal ion chelation. Employing a combination of these techniques provided deeper insight into the antioxidant potential of the tested extracts (Benzie and Strain, 1996). The findings were expressed as mg Trolox/g extract.

### 2.7. Antimicrobial activity

The antimicrobial activities of the *X. strumarium* extracts were determined by calculating minimum inhibitory concentration (MIC) values via broth dilution method as described by the Clinical and Laboratory Standards Institute (CLSI, 2008). Two gram-positive strains of cocci (*S. aureus* ATCC 25923, *E. faecalis* ATCC 51299), two gram-negative bacillus strains (*E. coli* ATCC 25922, *P. aeruginosa* ATT 27853), and one fungus (*C. albicans* ATCC 90028) were used in this study. Tryptic soy agar medium (OXOID, İstanbul, Türkiye) was used for the culturing of bacterial strains, while Sabouraud dextrose agar (OXOID) was used for the fungus. Microorganisms were incubated aerobically at 35 °C for 24–48 h. Bacterial inoculum was added to achieve a final density of 5 × 10^8^ CFU/mL in sterile saline (0.85% NaCl). In 96-well microplates with final inoculum concentrations of 1 × 10^5^ CFU/mL extracts (decreasing from 5000 to 90 μg/mL), positive controls (medium containing microorganisms alone) and negative controls (medium containing just plant extract) were added. Serial dilutions of the plant extracts were prepared for bacterial and fungal cells using Mueller Hinton Broth (OXOID) and RPMI 1640 (Thermo Fischer Scientific, İstanbul, Türkiye), respectively. MIC values, as the lowest concentration of the extract that completely inhibited microbial growth, were obtained after all inoculation plates had been cultured for 24–48 h at 35 °C.

### 2.8. Cell culture and cytotoxicity

The American Type Culture Collection (ATCC, Manassas, VA, USA) supplied the K562 (chronic myelogenous leukemia) and P3HR1 (Burkitt’s lymphoma) cell lines, which were grown in Iscove’s modified Dulbecco medium (IMDM) enriched with 10% fetal calf serum and 625 μl/L gentamycin as supplementation. Cells at a concentration of 10^5^ cells/mL were cultivated in 96-well plates and held at 37 °C in a moisture-controlled incubator with 5% CO_2_ throughout the experiment. Assessment of cytotoxicity was conducted by MTT assay (Mosmann, 1983). For this purpose, 10 μL of plant extract at varied concentrations (100 to 500 μg/mL) was added in triplicate to each well, with wells containing solely IMDM serving as the negative control. Subsequently, the cells were seeded at a density of 10^5^ per well and incubated for 72 h. The control wells were devoid of any extracts. A minimum of three distinct experiments were performed. Following the removal of the acidified media, wells were supplemented with 5 mg/mL MTT after the incubation phase. Subsequently, the samples underwent a 3-h incubation at a temperature of 37 °C. To dissolve the formazan crystals, each well was supplemented with 100 μL of acidic isopropanol alcohol (0.04 M HCl). The microplates remained in darkness overnight and then the samples were analyzed using an ELISA multiwell spectrophotometer at 540 nm with a reference wavelength of 620 nm. The half-maximal inhibitory concentration (IC_50_) values were calculated based on means and standard deviations.

### 2.9. Western blot analysis

Cells (45 mL; 10^5^ cells/mL culture medium) were treated with the IC_50_ values of *X. strumarium* extracts (acetone, methanol, and ethanol) for 72 h to analysis apoptotic protein expression. Collected cells were washed twice with ice-cold PBS. After 1 h of lysis buffer treatment (50 mM Tris-HCl of pH 7.5, 250 mM NaCl, 5 mM EDTA, 1 mM EGTA, 1 mM NaF, 1 mM phenylmethylsulfonyl fluoride, 1 mM dithiothreitol, 20 μg/mL leupeptin, 20 μg/mL aprotinin, 0.1% Triton X-100, and 1% SDS), with a short period of sonication, lysates were centrifuged at 13,000 × *g* for 10 min at 4 °C. To determine the protein levels in each sample, a protein assay kit was utilized (Bio-Rad, Hercules, CA, USA). Electrophoresis of an identical amount of samples (50 μg of protein) was performed on SDS-polyacrylamide gel (10% or 12%). After electrophoresis, blots of protein were transferred to a nitrocellulose membrane. The membrane was blocked by 5% bovine serum albumin in TBST solution and then the blocking solution was incubated overnight at 4 °C with primary antibodies against caspase-3, caspase-8, cytochrome c, and β-actin (Abcam, Cambridge, MA, USA). The membrane was incubated with alkaline phosphatase (AP)-conjugated antirabbit secondary antibody and diluted with TBST solution (1:10,000) at room temperature for 1 h after washing with TBST solution (10 mM Tris-HCl of pH 7.5, 100 mM NaCl, and 0.1% Tween 20) three times. Colorimetric AP staining methodology (Bio-Rad BCIP/NBT substrate) was used to visualize the identified protein signals as recommended by the manufacturer. ImageJ software (National Institutes of Health, Bethesda, MD, USA) was utilized for band intensity comparisons and quantification with respect to β-actin expression levels (Sergazy et al., 2022).

### 2.10. DNA fragmentation (diphenylamine assay)

The diphenylamine assay was used for quantitative estimation of DNA fragmentation (Gercel-Taylor, 2005). Collected cells were suspended in TE lysis buffer (0.5% Triton X-100, 5 mM Tris, 20 mM EDTA, pH 8.0) for 30 min at 4 °C. DNA fragments and damage were isolated from the supernatant fractions by centrifugation. TE buffer was used to resuspend the pellets. The pellets and supernatants were mixed with 10% TCA and the mixture was precipitated overnight at 4 °C. To remove proteins, the precipitates were centrifuged at 4 °C, boiled at 100 °C for 15 min, and then resuspended in 5% TCA. Diphenylamine (320 μL) was added and the color took 4 h to develop at 37 °C. At 600 nm, the absorbance was measured with an ELISA multiwell spectrophotometer. The ratio of DNA content in the supernatant to that in the pellet and supernatant was used to compute the percentage of total DNA.

### 2.11. Data analysis

The results of triplicate tests were analyzed and expressed as mean ± standard deviation (n = 3) using Microsoft Excel (2013 version; Microsoft Corp., Redmond, WA, USA). Student’s t-test was used to assess the statistical significance of the findings and values of p < 0.05 were deemed statistically significant.

## 3. Results

Extract yields (w/w) were determined for the acetone (0.92%), methanol (8.36%), and ethanol (3.15%) extracts, respectively, obtained from *X. strumarium*. Qualitative phytochemical analysis of different extracts showed the existence of alkaloids, saponins, terpenoids, and glycosides in all extracts, whereas flavonoids and tannins were detected only in the acetone and ethanol extracts (Table 1).

The TPCs of different *X. strumarium* extracts are shown in Table 2. The highest TPC values were detected in the acetone extract (454.54 ± 4.32 mg GAE/g). Three different procedures, including DPPH, ABTS, and FRAP assays, were used to determine the antioxidant activity of the *X. strumarium* extracts. All three methods showed that the acetone extract had the highest activity levels (442.81 ± 5.21, 234.34 ± 5.25, and 337.70 ± 6.14 mg Trolox/g, respectively), although the values were comparable to those of the other two extracts. The methanol and ethanol extracts had similar antioxidant activity levels (Table 2).

**Figure 1:**
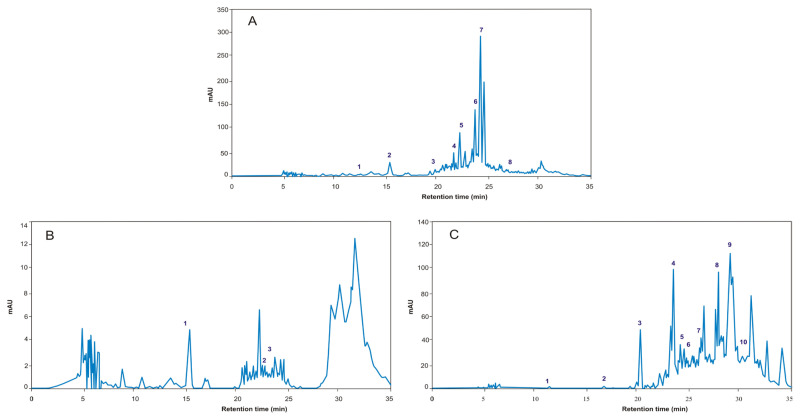
HPLC-DAD chromatograms of *X. strumarium*: **A**) acetone extract containing protocatechuic acid (1), chlorogenic acid (2), epigallocatechin gallate (3), epicatechin gallate (4), p-coumaric acid (5), ferulic acid (6), rosmarinic acid (7), luteolin (8), naringenin (9), and kaempferol (10); **B**) methanol extract containing chlorogenic acid (1), p-coumaric acid (2), and ferulic acid (3); **C**) ethanol extract containing (1) protocatechuic acid, (2) chlorogenic acid, (3) vanillic acid, (4) caffeic acid, (5) rutin, (6) p-coumaric acid, (7) ferulic acid, and (8) resveratrol.

HPLC-DAD analysis was used to identify the primary constituents in the extracts, employing 16 standards. Compounds were identified based on their absorbance spectra and retention times. Figure 1 displays the HPLC chromatographic profiles of the phenolics in the acetone, methanol, and ethanol extracts. For the detected phenolic substances (Table 3), the highest level of epigallocatechin gallate was 13.60 ± 0.03 mg/g in the acetone extract.

The effects of the methanol and ethanol extracts were similar against gram-negative bacteria (*E. coli* and *P. aeruginosa*; MIC: 1250 μg/mL) and a gram-positive bacterium (*S. aureus*; MIC: 2500 μg/mL). The MIC values of the acetone extract against *C. albicans*, *S. aureus*, *E. faecalis*, *E. coli*, and *P. aeruginosa* were 9, 1250, 39, 156, and 39 μg/mL, respectively (Table 4).

**Figure 2:**
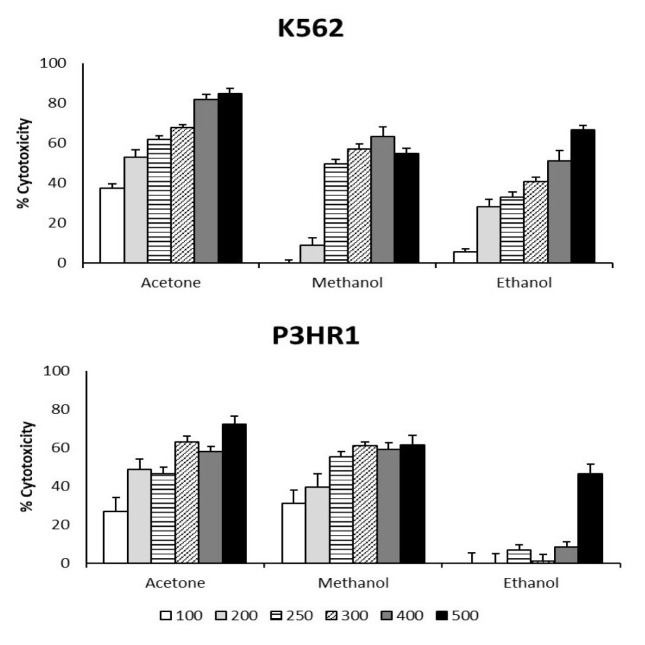
Cytotoxic activity of *X. strumarium* extracts at various concentrations (100–500 μg/mL) against K562 and P3HR1 cell lines.

Figure 2 presents the results of the cytotoxicity assay performed in the present study. The IC_50_ values of the acetone, methanol, and ethanol extracts against the K562 cell line were 180.12 ± 0.9, 298.25 ± 0.6, and 410.23 ± 0.1 μg/mL, respectively, while they were 221.81 ± 0.4, 380.62 ± 0.8, and >500 μg/mL, respectively, for the P3HR1 cell line. The IC_50_ values of the acetone extract were lower compared to the other extracts.

**Figure 3:**
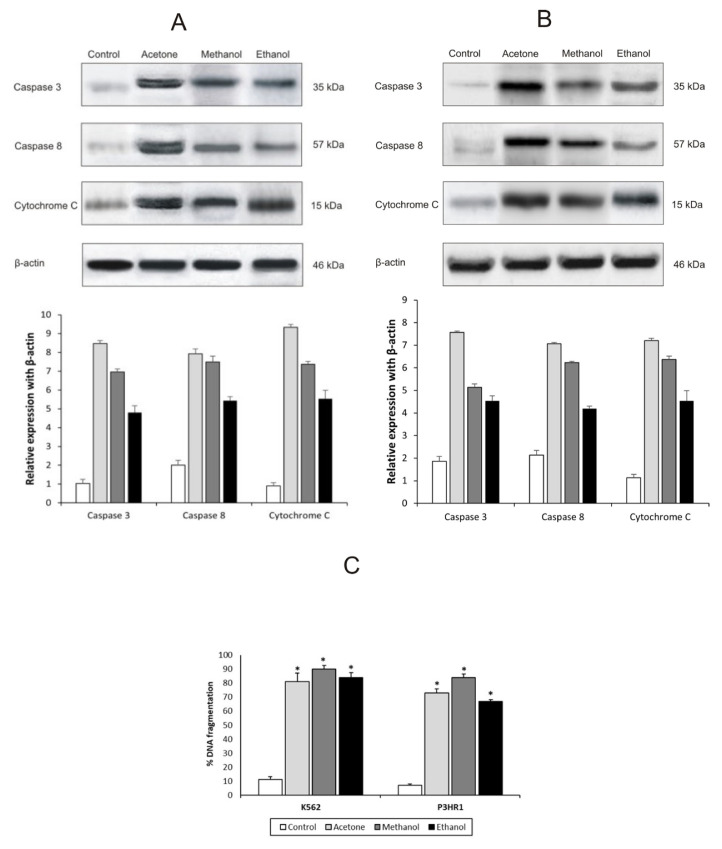
Caspase expression levels by western blotting for K562 **(A)** and P3HR1 **(B)** cell lines after *X. strumarium* treatment. **C)** Percentage of DNA fragmentation achieved by *X. strumarium* extracts with IC_50_ values for K562 and P3HR1 cells.

To investigate the molecular mechanism behind the apoptosis process, the expression levels of caspase-3, caspase-8, and cytochrome c, which are essential for apoptotic signaling, were analyzed using western blot analysis (Figures 3A and 3B). β-Actin was the loading control for the western blotting and was used for calculating protein expression levels. The results demonstrated that, in comparison to the control, treatment with *X. strumarium* extracts increased the expression levels of cytochrome c, caspase-3, and caspase-8 in varying ways.

Figure 3C illustrates the effects of the *X. strumarium* extracts on DNA fragmentation. The percentage of DNA fragmentation of all extracts increased significantly (p < 0.05) in both investigated cell lines.

## 4. Discussion

Alkaloids, flavonoids, tannins, triterpenoids, glycosides, and saponins have all been found in various parts of *X. strumarium* in previous studies. Extraction solvents have an obvious impact on the nature and quantity of extracted active compounds from plants, and the presence of these compounds may be the main cause of biological activity. In this study, acetone, methanol, and ethanol were selected as extraction solvents due to their varying polarities, allowing for efficient extraction of a wide range of bioactive compounds, including both polar and nonpolar phytochemicals. The environmental variables of altitude, illumination, temperature, humidity, and soil type vary depending on the location of the collected plant. These variations in environmental factors affect the types and concentrations of metabolites in different plants of the same species, which affect the quality and therapeutic effects of medicinal plants in turn (Dong et al., 2011).

It is known that free radicals are involved in a number of illnesses, such as infectious ailments and cancers (Pohanka, 2013; Phaniendra et al., 2015; Xu et al., 2017). The antioxidant activity of plant extracts is associated with their various bioactive compounds in line with their ability to scavenge free radicals (Saito et al., 2008). In this study, the antioxidant capacity of *X. strumarium* extracts was assessed utilizing FRAP, DPPH, and ABTS assays. It was observed from these assays that the *X. strumarium* extracts with the highest phenolic contents displayed high antioxidant activities. These findings are in agreement with previous publications reporting that extraction with low-polarity solvents resulted in the highest TPCs (Kamboj et al., 2014; Ly et al., 2021). The capacity of extracts to donate electrons and react with free radicals to produce more stable compounds and break the cycle of radical reactions is suggested by this ability. The specific solvent’s polarity and its capacity to extract particular classes of compounds are probably the causes of the differing concentrations of phenolic compounds in various extracts. For instance, compared to the ethanol and methanol extracts, the acetone extract of the present study had larger quantities of certain phenolic compounds. Considering the varied polarities of the solvents used in the extraction of *X. strumarium*, p-coumaric acid, chlorogenic acid, and ferulic acid were present in all extracts. HPLC-DAD revealed the highest phenolic contents in the acetone extract. As a result, the chromatographic and spectroscopic data appear to support each other.

Large numbers of the diverse phytochemicals obtained from plant extracts show high levels of effectiveness against infections. However, despite the extensive research conducted every year on plants, there are still many plants waiting to be analyzed, some of which may constitute important sources of antimicrobial agents. The focus of many researchers is now shifting to plant-derived medications due to antibiotic resistance, side effects, and the exorbitant costs associated with the development of synthetic drugs (AlSheikh et al., 2020). In a previous study, a methanolic extract from the aerial parts of *X. strumarium* was found to be active against *Proteus vulgaris*, *S. aureus*, *Bacillus subtilis*, *C. albicans*, and *C. pseudotropicalis* (Jawad et al., 1988). Leaf extracts of *X. strumarium* showed strong antibacterial activity against *Salmonella* serovar Typhimurium, *E. coli*, *P. aeruginosa*, *S. aureus*, and *C. perfringens*. *S. aureus* and *C. perfringens* were more sensitive to nonpolar fractions than polar fractions; however, there was no discernible variation in the extracts’ antibacterial effects among the remaining bacteria (Scherer et al., 2009). In another study, the same researchers reported that hydrodistilled and supercritical leaf extracts of *X. strumarium* inhibited the growth of gram-positive and gram-negative bacterial strains (Scherer et al., 2010). In a different investigation, a methanolic leaf extract of *X. strumarium* showed efficacy against methicillin-susceptible and methicillin-resistant *S. aureus* (Rad et al., 2013). A methanolic extract prepared from the fruit of *X. strumarium* demonstrated strong antibacterial effects against *S. aureus* and *Streptococcus agalactiae* (Ingawale et al., 2018). In summary, the acetone extract exerted more significant antimicrobial activity than the methanol or ethanol extracts. This activity can be explained by the existence of more active compounds that are soluble in nonpolar solvents.

Conventional cytotoxic chemotherapy kills cancer cells by inducing apoptosis but unfortunately also has side effects and long-term sequelae; furthermore, many tumors become resistant (Garber, 2005). As a result, medications that restore apoptotic pathways may be useful in treating cancer. Ethnopharmacological expertise is useful in directing the search for plants with cytotoxic potential. The acetone extract of *X. strumarium* exhibited the maximum cytotoxicity against both the K562 and P3HR1 cell lines, but all three extracts induced cell cytotoxicity in a concentration-dependent manner.

One of the goals of this study was to see how the *X. strumarium* extracts affected the K562 and P3HR1 cell lines. The results confirmed the dose-dependent inhibition of the viability of both cell lines in response to the *X. strumarium* extracts. The acetone extract had higher cytotoxicity in comparison to the other extracts, which might be due to the fact that acetone is less polar than the other solvents used in extraction, leading to better extraction for secondary metabolites that are active against leukemic cell lines (Ngo et al., 2017). Alkaloids, flavonoids, glycosides, tannins, terpenoids, and saponins were previously discovered in different extracts of *X. strumarium* (Ramírez-Erosa et al., 2007; Wahab et al., 2012). The presence of these components could be the primary cause of the biological activity of the plant.

One of the most important cell death mechanisms is apoptosis. Caspases and DNA fragmentation are two of the primary processes of apoptotic cell death. Intrinsic pathways such as the mitochondrial pathway and extrinsic pathways such as the death receptor pathway are used to cause apoptosis. By the intrinsic mechanism, cytochrome c is released from the mitochondria, while caspase-8 is important in the extrinsic mechanism. In both types of pathways, caspase-3 is present (Fulda and Debatin, 2006). In the current study, western blotting was used to explore the expression levels of all three proteins in order to learn more about the molecular process of apoptosis. As a result, the expression of cytochrome c was found to have increased in both K562 and P3HR1 cells. This shows that apoptosis was triggered by a mitochondrial intrinsic mechanism, which agrees with the results of a previous study of *Allium sativum*-derived small extracellular vesicles on the mechanistic level (Ünsal et al., 2024). Mitochondrial activity and reactive oxygen species engage in apoptosis under both normal and pathological conditions, and after the mitochondrial membrane’s potential is lost, reactive oxygen species are produced (Zamzami et al., 1995). It is uncommon for mitochondria to be both a source and a target of reactive oxygen species. The cytochrome c generated by mitochondria, mediated by either direct or indirect reactive oxygen species activity, activates caspases (Simon et al., 2000). DNA fragmentation was found to differ significantly (p < 0.05) among the three extracts in contrast to the controls for the cell lines used in the present study. The DNA fragmentation in both cell lines may have been triggered by a process that included an apoptotic pathway (Carvalho et al., 2012). DNA fragmentation results from extract-induced apoptosis by increased caspase activation through both intrinsic and extrinsic pathways, which leads to cytotoxicity in turn.

The findings of this investigation confirm the potential of *X. strumarium* for use in healthcare thanks to the bioactive compounds that it contains. The purification of extracts, identification of active components, and establishment of quality standards are necessary.

## Figures and Tables

**Table 1:** Phytochemical screening of *X. strumarium* extracts. Key: −, Absent; +, Present.

Phytochemicals	Acetone	Methanol	Ethanol
Alkaloids	+	+	+
Flavonoids	+	−	+
Saponins	+	+	+
Terpenoids	+	+	+
Tannins	+	−	+
Glycosides	+	+	+

**Table 2:** Total phenolic and flavonoid contents and antioxidant activities of the extracts of *X. strumarium*.

Extracts	TPC (mg GAE/g)	TFC (mg Catechin/g)	DPPH (mg Trolox/g)	ABTS (mg Trolox/g)	FRAP (mg Trolox/g)
Acetone	454.54 ± 4.32^a^	78.94 ± 2.25^a^	442.81 ± 5.21^a^	234.34 ± 5.25^a^	337.70 ± 6.14^a^
Methanol	298.45 ± 2.76^b^	47.16 ± 1.06^b^	317.42 ± 3.49^b^	111.82 ± 0.72^b^	209.46 ± 0.41^b^
Ethanol	302.68 ± 3.16^b^	50.46 ± 1.53^b^	303.16 ± 5.81^b^	118.34 ± 1.91^b^	220.35 ± 4.17^b^

There are no significant differences (p > 0.05) between extracts that share the same superscripted letter in the same column.

**Table 3:** Amounts of phenolic substances in *X. strumarium* extracts (mg/g).

Phenolic compound	Acetone	Methanol	Ethanol
Protocatechuic acid	0.29 ± 0.01	nd	0.72 ± 0.01
Vanilic acid	nd	nd	0.53 ± 0.02
Epigallocatechin gallate	13.60 ± 0.03	nd	nd
Epicatechin gallate	13.53 ± 0.02	nd	nd
Naringenin	12.18 ± 0.21	nd	nd
Chlorogenic acid	0.30 ± 0.01	2.34 ± 0.07	8.33 ± 0.04
Caffeic acid	nd	nd	0.48 ± 0.01
p-coumaric acid	0.58 ± 0.01	0.10 ± 0.01	5.63 ± 0.04
Ferulic acid	1.50 ± 0.01	0.26 ± 0.01	11.15 ± 0.02
Rosmarinic acid	3.65 ± 0.01	nd	nd
Resveratrol	nd	nd	0.24 ± 0.01
Routine	nd	nd	6.61 ± 0.06
Luteolin	2.84 ± 0.01	nd	nd
Campherol	1.19 ± 0.01	nd	nd

**Table 4:** MICs of *X. strumarium* extracts against different microbial strains.

Microorganisms	Evaluated plant extracts (MIC μg/mL)		
Bacteria	Acetone	Methanol	Ethanol
*S. aureus*	1250	2500	2500
*E. faecalis*	39	156	625
*E. coli*	156	1250	1250
*P. aeruginosa*	39	1250	1250
**Fungi**			
*C. albicans*	9	625	1250
